# Supplementary figures and images for: Comparative genomics reveals electron transfer and syntrophic mechanisms differentiating methanotrophic and methanogenic archaea
Source: PLoS Biol. 2022 Jan 5;20(1):e3001508. doi: 10.1371/journal.pbio.3001508 (PMC9012536; doi:10.1371/journal.pbio.3001508)

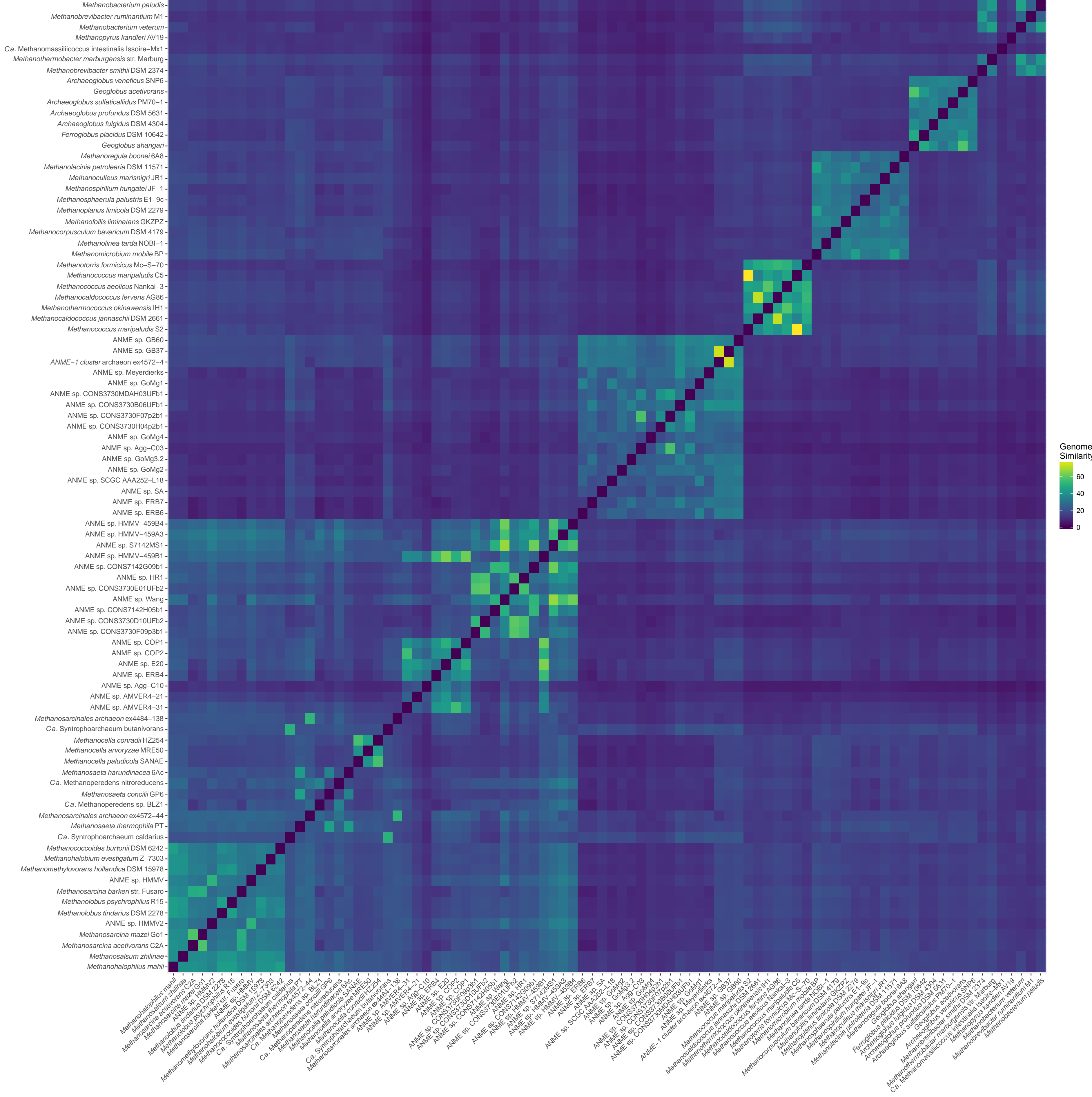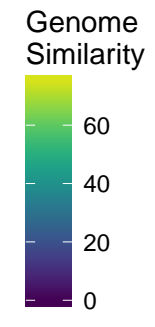

Supplement: S1 Fig — GS between methanogen and ANME genomes reported here. Details of similarity calculations can be found below in the Materials and methods. ANME, anaerobic methanotrophic; GS, genome similarity. (PDF) [file pbio.3001508.s001.pdf]

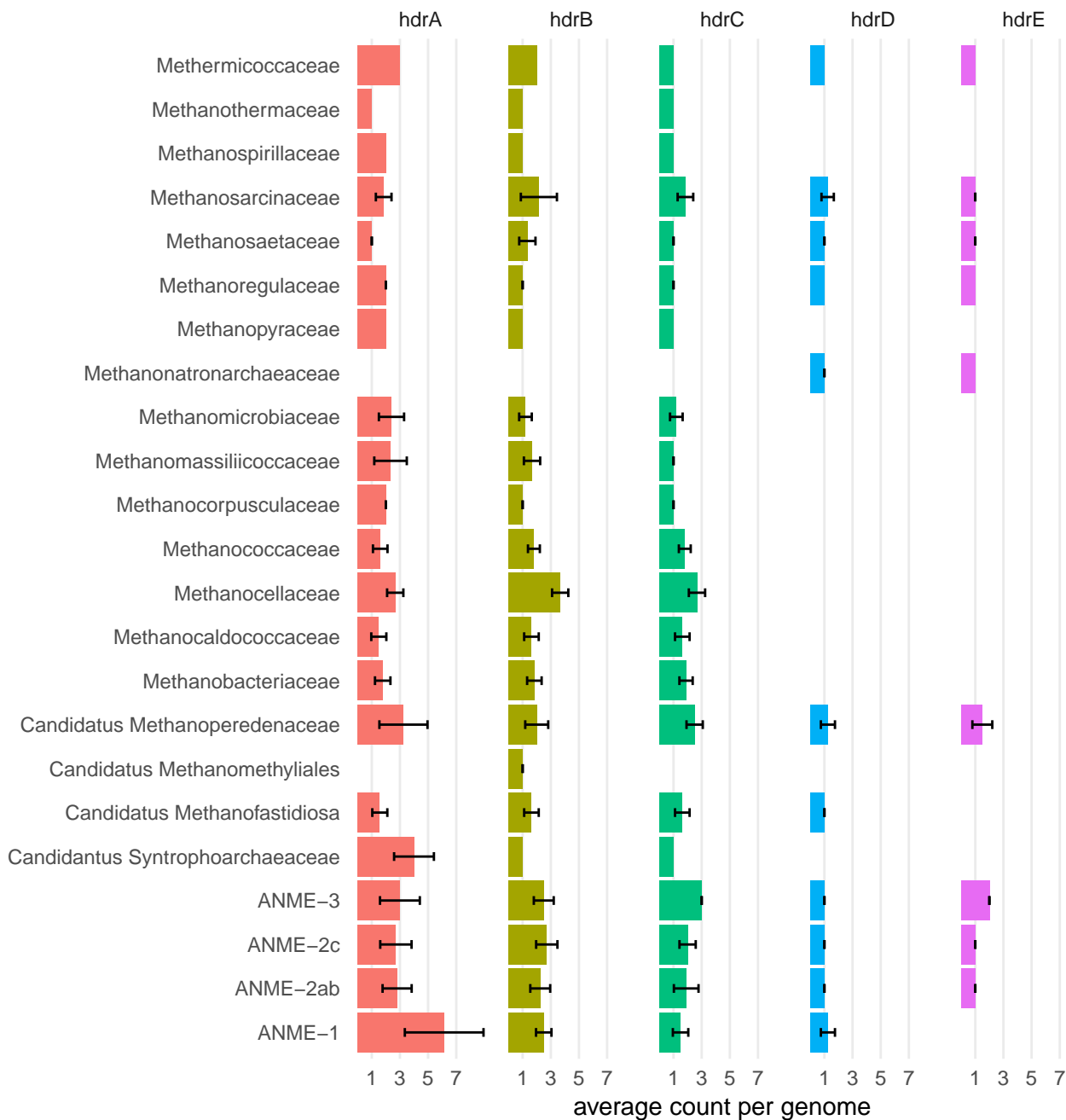

Supplement: S9 Fig — Most ANME and methanogen genomes contain 1–2 copies of hdrABC genes; however, ANME-1 have a much greater abundance of hdrA homologs that are not accompanied by an increase in the number of hdrB or C homologs. ANME, anaerobic methanotrophic. (PDF) [file pbio.3001508.s009.pdf]

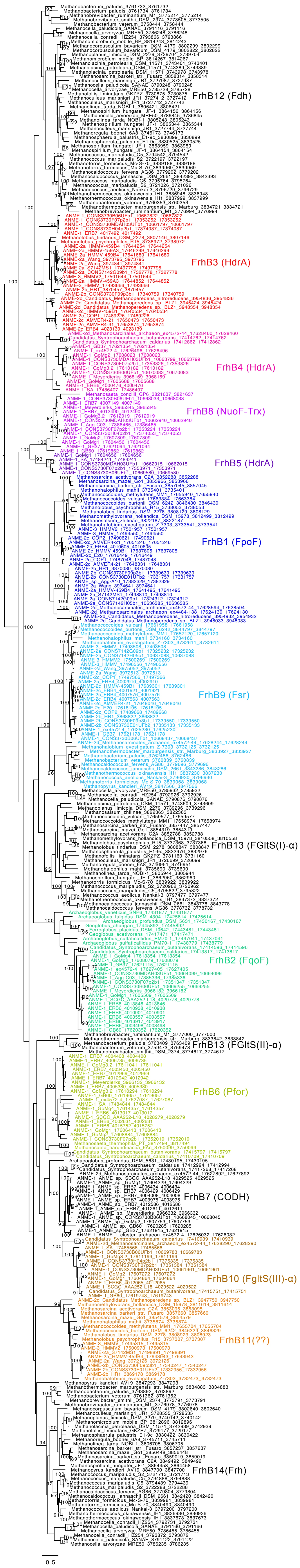

Supplement: S16 Fig — FrhB paralogs are labeled as in Fig 16. Only branch support values >90% are shown for clarity. Tree scales represent substitutions per site. Tree construction parameters are found in the Materials and methods section. Alignment and tree files can be found in S1 Data. (PDF) [file pbio.3001508.s016.pdf]
